# Effectiveness of Thumb Sleeve Distraction and Pop‐It Therapeutic Play in Reducing Dental Anxiety and Pain in Children Aged 4–6 Years Undergoing Mandibular Alveolar Nerve Block: A Randomized Controlled Trial

**DOI:** 10.1155/ijod/9319639

**Published:** 2026-06-23

**Authors:** Alaa Mohammed Snobar, Chaza Nader Kouchaji, Mohammed N. Al-Shiekh

**Affiliations:** ^1^ Department of Pediatric Dentistry, Faculty of Dentistry, Damascus University, Damascus, Syria, damascusuniversity.edu.sy

**Keywords:** behavioral management, dental anxiety and pain, local anesthesia, sensory distraction, Tell–Show–Do, visual distraction

## Abstract

**Background:**

Distraction is a safe and effective technique that diverts a child’s attention from anxiety‐provoking stimuli. This study aimed to evaluate the efficacy of thumb sleeves as a visual distraction and Pop‐It toys as therapeutic play in reducing dental anxiety and pain in children aged 4–6 years undergoing mandibular alveolar nerve block.

**Materials and Methods:**

This double‐blind trial included 100 children aged 4–6 years, randomly allocated into four parallel groups: Group I (Control)—Tell–Show–Do (TSD) alone; Group II—TSD with visual distraction (thumb sleeve); Group III—TSD with sensory distraction (Pop‐It toy); Group IV—TSD with both visual and sensory distractions. Pulse rate was measured at baseline (T0), after behavior management (T1), and 2 min after local anesthesia (T3). Dental anxiety was assessed using the Facial Image Scale at T0 and T3, and pain was measured using the Face, Legs, Activity, Cry, Consolability (FLACC) scale at T2. Statistical significance was set at *p*  < 0.05. Kolmogorov–Smirnov was used to test the normal distribution, and the Kruskal–Wallis test was used to compare the studied groups.

**Results:**

Anxiety and pain scores varied among the groups. Based on pulse rate, Group III (TSD with sensory distraction) showed the lowest anxiety after behavior management, while Group I (TSD alone) showed the highest. After 2 min of anesthesia, Group IV (combined visual and sensory distraction) demonstrated the lowest anxiety, whereas Group II showed the highest. According to the Facial Image Scale, Group IV recorded the lowest anxiety and Group III the highest at T3. In terms of pain (FLACC), Group IV showed the lowest scores, while Group I recorded the highest during anesthesia administration. However, the Kruskal–Wallis test revealed no statistically significant differences between groups in pulse rate, Facial Image Scale, or FLACC scores (*p* > 0.05).

**Conclusion:**

Although no statistically significant differences were found between the studied groups, the use of distraction techniques combined with TSD showed a trend toward greater reduction in dental anxiety and pain compared with TSD alone. These approaches may be considered as supportive behavior management tools during inferior alveolar nerve block (IANB) in young children.

**Trial Registration:** ClinicalTrials.gov identifier: NCT06976047

## 1. Introduction

Dental anxiety is a significant challenge in pediatric dentistry, often affecting children’s cooperation and overall treatment outcomes [1]. It is characterized by negative thoughts, fear, and a perceived loss of control, commonly arising in response to invasive procedures such as injections, high‐speed handpieces, and sharp instruments [2]. The prevalence of dental anxiety among children ranges from 23.9% to 52%, depending on the age group [3, 4].

This condition can have substantial consequences, including neglected oral hygiene, increased risk of dental caries, and a range of physical, emotional, cognitive, and behavioral effects. These may include heightened pain perception, mood disturbances, social limitations, sleep problems, and reduced self‐esteem, which may persist in the long term [5].

Pain, whether associated with actual or potential tissue damage or occurring in the absence of identifiable injury, further complicates dental care and is closely linked to the experience of anxiety in children [6].

To address these challenges, behavior management is considered a cornerstone of pediatric dental care. The American Academy of Pediatric Dentistry defines behavior management as a continuous interaction between the dentist, dental team, and patient, emphasizing communication, education, and safety [7]. These strategies are broadly classified into pharmacological and nonpharmacological approaches [8].

Among nonpharmacological techniques, distraction and therapeutic play are widely used. Distraction is a safe and effective method that diverts the child’s attention away from anxiety‐provoking stimuli, thereby reducing perceived pain [9, 10].

Modern distraction approaches in pediatric dentistry include techniques such as magic tricks (sleight of hand), which apply principles of cognitive misdirection to redirect attention away from anxiety‐provoking stimuli toward more engaging and positive visual experiences [11]. Emerging evidence indicates that such magic‐based interventions may enhance cooperation and reduce anxiety in children during dental treatment [12–14]. However, the current literature is limited by heterogeneity in study designs and outcome measures and is largely focused on single‐modality interventions [15]. Moreover, there remains insufficient evidence regarding their effectiveness in younger children and during invasive dental procedures, highlighting the need for further well‐designed clinical trials.

In a similar context, therapeutic play has been shown to support behavioral modification, improve cooperation, and facilitate cognitive and emotional development while also enhancing communication and interaction with the surrounding environment. Although sensory‐based interventions such as Pop‐It toys have demonstrated beneficial effects in reducing anxiety in medical settings, their application and effectiveness in pediatric dental practice remain underexplored [16, 17].

Despite the increasing incorporation of these nonpharmacological strategies, evidence supporting their effectiveness in pediatric dentistry is still limited, particularly in young children undergoing invasive procedures such as the inferior alveolar nerve block (IANB). Therefore, the current study aimed to evaluate the efficacy of thumb sleeves as a visual distraction technique and Pop‐It toys as therapeutic play in reducing dental anxiety and pain among children aged 4–6 years undergoing mandibular alveolar nerve block.

## 2. Materials and Methods

### 2.1. Ethical Approval and Study Design

This study was conducted in accordance with the ethical principles outlined in the Declaration of Helsinki and adhered to the CONSORT guidelines [18, 19]. The Ethics Committee of Damascus University approved this study (Approval Number 3176/22042024).

Gender, ethnicity, and socioeconomic background were not used as criteria for inclusion or exclusion in this study. Before recruitment, all study procedures were explained in detail to the children’s parents or legal guardians, and written informed consent was obtained prior to participation.

This randomized controlled clinical trial was conducted in the Department of Pediatric Dentistry, Faculty of Dentistry, Damascus University, over 8 months from December 2024 to July 2025.

### 2.2. Participants Recruitment

The required sample size was estimated using 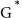
Power software version 3.1.9.4 (Heinrich Heine University, Düsseldorf, Germany), informed by the methodology of Kothari et al. [12]. A total of 100 participants (25 in each of the four groups) was calculated to provide 80% power (1 − β) at a 5% significance level (*α* = 0.05) to detect a small‐to‐moderate effect size (*f* = 0.6) [20, 21]. To further validate the effect size, a pilot study was performed with 10 children prior to the main trial [22].

Overall, 106 children who attended the Department of Pediatric Dentistry, Damascus University, were assessed against predefined eligibility criteria. Of these, 100 fulfilled the criteria and were enrolled in the trial. Eligible participants were randomly assigned to four study groups using block randomization with a 1:1:1:1 allocation ratio to ensure an equal group distribution.

Children aged 4–6 years with no previous dental experience, who demonstrated a positive or definitely positive rating on Frankl behavior rating scale and required an IANB, were included.

Exclusion criteria comprised children with systemic or mental disorders, those who had received sedatives or analgesics within 8 h prior to the dental visit, and children with contraindications to regional anesthesia.

Children were allocated to four study groups: Group I (Control): Anesthesia procedures were performed using the Tell–Show–Do (TSD) approach alone. Group II: anesthesia procedures were combined with TSD and visual distraction using magic tricks (thumb sleeve). Group III: anesthesia procedures were combined with TSD and sensory distraction using a Pop‐It toy. Group IV: anesthesia procedures were combined with TSD and both visual (thumb sleeve) and sensory (Pop‐It toy) distractions.

### 2.3. Blinding and Randomization

In this study, both the children and the data analyst were blinded to group assignments. The children were not informed of their group allocation or the specific objectives of the study, and group identities remained concealed during data analysis. However, due to the nature of the interventions, the operator and outcome assessor were not blinded.

After obtaining parental consent, 100 eligible children were randomly assigned to study groups using the www.randomizer.org website, with each participant allocated a unique random number. Block randomization was performed via www.randomization.com to ensure equal group sizes, resulting in four groups of 25 children each, with a 1:1:1:1 allocation ratio.

### 2.4. Primary Outcomes Measure

– Pulse rate: A fingertip pulse oximeter was placed on the left index finger to measure pulse rate. Measurements were recorded at the following time points: T0, before treatment; T1, after the application of the behavior management technique; and T3, after 2 min of administration of anesthesia.
– Facial Image Scale: FIS measurements were recorded at two time points: T0, while the child was at rest before treatment, and T3, after 2 min of administration of anesthesia.
– Face, Legs, Activity, Cry, Consolability (FLACC) scale: The scale assesses five behavioral domains: activity, crying, leg movement, facial expression, and consolability. Each domain is scored from 0 to 2, yielding a total score ranging from 0 to 10, where 0 indicates no pain and 10 indicates severe pain [23, 24]. This scale was used at one time point: T2, during the administration of anesthesia.

### 2.5. Intervention

– Group I (Control group): Verbal distraction was used in combination with the TSD strategy during the administration of the IANB.

To reduce fear and enhance understanding, the procedure was first explained to the child in simple, developmentally appropriate language (“Tell”). This was followed by a demonstration of the materials and sensations using a model or a nonthreatening demonstration on the child’s hand (“Show”). Once the child appeared comfortable, the nerve block was administered exactly as explained (“Do”), ensuring consistency between explanation and action.

Verbal distraction was maintained throughout the procedure through nonprocedural conversation, positive reinforcement, and the use of calming, encouraging language.

– Group II (visual distraction group): To implement visual distraction, the child was encouraged to engage with the activity by attempting to “catch” the light before anesthesia.

The dentist performed several hand motions while wearing a thumb sleeve equipped with a light, which was controlled by pressing a battery‐operated switch. The child was instructed to follow the light, creating the illusion that it was being passed from one hand to the other and then “swallowed” into the mouth.

The IANB was administered once the child appeared comfortable and focused on the activity.

– Group III (sensory distraction group): A Pop‐It toy was provided to the child as a sensory distraction during the administration of local anesthesia.

Throughout the procedure, the child was encouraged to actively engage with the toy through brief instructions on its use and a short explanation of the play activity.

– Group IV (visual and sensory distraction): The interventions were applied in a sequential manner rather than simultaneously. The procedure began with a visual distraction using a thumb sleeve light, where the dentist performed several hand movements to create the illusion of the light being transferred between hands and then “swallowed” into the mouth, while the child was instructed to follow and “catch” the light. After the child became engaged with the visual task, a Pop‐It toy was introduced as a sensory distraction during the administration of local anesthesia. The child was encouraged to interact with the toy through brief instructions and simple explanations of its use throughout the procedure.

The IANB was administered once the child appeared comfortable and focused on the combined activities.

The following procedures were applied to all study groups in accordance with Alkhouli et al. [25] study. Prior to administration of the IANB, topical anesthesia was performed using 20% benzocaine gel (Darby, DDS, LLC), applied to the dried oral mucosa for 1 min. The IANB was administered using a 27‐gauge, 16‐mm needle; however, the needle gauge and length were adjusted according to the child’s age and anatomical considerations using either a long or short, 25‐ or 27‐gauge needle.

The thumb of the clinician was positioned on the occlusal surfaces of the molars, with the ball of the thumb on the retromolar fossa and the tip on the internal oblique ridge. On the opposite side of the dental arch, the syringe barrel was placed between the primary teeth. Following initial tissue penetration, a small quantity of the anesthetic was deposited. Subsequently, up to one‐quarter of a cartridge was gradually administered as the needle was advanced in 4 mm increments. After each negative aspiration, the needle was further advanced until a bony contact was achieved, with an average depth of ~15 mm.

A volume of 1 mL of 2% lidocaine with 1:200,000 epinephrine (Dentsply Sirona, USA) was administered around the inferior alveolar nerve.

### 2.6. Statistical Analysis

Descriptive statistics, including frequencies, percentages, means, standard deviations, and medians, were calculated for demographic and baseline variables. A *p*‐value < 0.05 was considered statistically significant for all tests. All statistical analyses were performed using SPSS version 25.0 (IBM Corp., Armonk, NY, USA).

The normality of distribution for numerical variables (age, pulse rate, the Facial Image Scale, and FLACC scale) was evaluated using the Kolmogorov–Smirnov test. As the data were not normally distributed (*p*‐value < 0.001), nonparametric tests were utilized for comparison analysis.

The gender distribution discrepancies between the study groups were assessed using the chi‐square test. In addition, to analyze age differences between the study groups, the Kruskal–Wallis test was used.

At each procedural time point, the Kruskal–Wallis was also used to assess intergroup differences in FLACC scale, Facial Image Scale scores, and pulse rate. If the Kruskal–Wallis test revealed significant differences, pairwise comparisons were conducted using the Mann–Whitney *U* test.

## 3. Results

**Figure 1:**
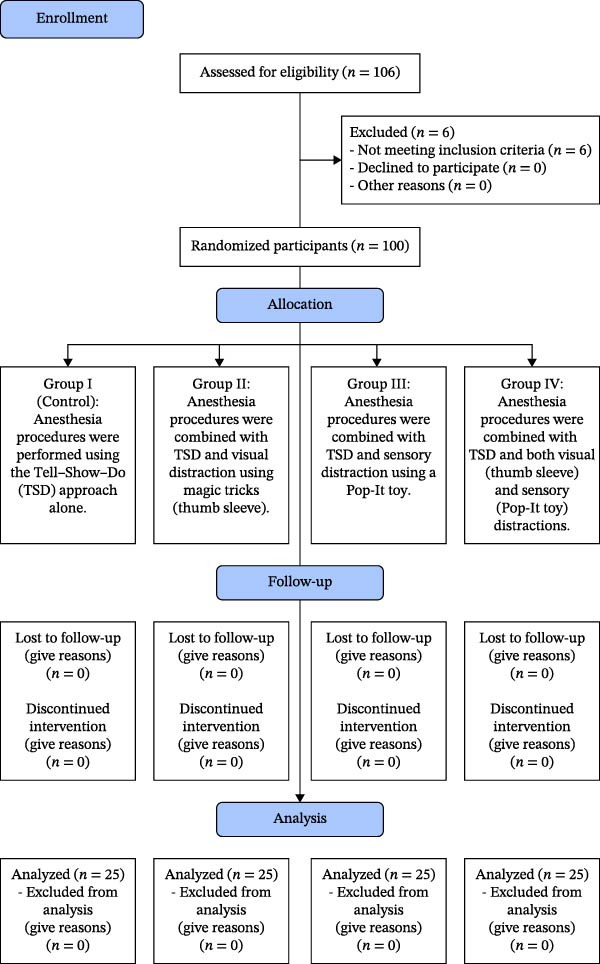
CONSORT diagram.

The CONSORT flow diagram is presented in Figure 1. Each study group comprised 25 children, yielding a total sample of 100 participants, of whom 44 were male (44%) and 56 were female (56%). Chi‐square analysis indicated no statistically significant difference in the gender distribution among the groups (*p*‐value = 0.312) (Table 1).

**Table 1:** Gender distribution of children across study groups and chi‐square test results.

	Male		Female		Total		*p*‐Value
	*N*	%	*N*	%	*N*	%	
Group I	10	10.0	15	15.0	25	25.0	0.312
Group II	15	15.0	10	10.0	25	25.0	
Group III	10	10.0	15	15.0	25	25.0	
Group IV	9	9.0	16	16.0	25	25.0	
Total	44	44.0	56	56.0	100	100.0	—

*Note:* Group I (Control): anesthesia procedures were performed using the Tell–Show–Do (TSD) approach alone. Group II: anesthesia procedures were combined with TSD and visual distraction using magic tricks (thumb sleeve). Group III: anesthesia procedures were combined with TSD and sensory distraction using a Pop‐It toy. Group IV: anesthesia procedures were combined with TSD and both visual (thumb sleeve) and sensory (Pop‐It toy) distractions.

Abbreviations: *N*, number of cases; %, percentage.

The participants’ ages ranged from 4 to 6 years across all groups, with an overall mean age of 5.34 ± 0.70 years. The Kruskal–Wallis test revealed no statistically significant difference in age distribution among the groups (*p*‐value = 0.326) (Table 2).

**Table 2:** Descriptive statistics of children’s age across study groups and Kruskal–Wallis test results.

	Minimum	Maximum	Median	Mean	Std. deviation	*p*‐Value
Group I	4.00	6.00	6.00	5.44	0.651	0.326
Group II	4.00	6.00	6.00	5.36	0.757	
Group III	4.00	6.00	5.00	5.12	0.726	
Group IV	4.00	6.00	6.00	5.44	0.651	
Total	4.00	6.00	5.00	5.34	0.699	—

*Note:* Group I (Control): anesthesia procedures were performed using the Tell–Show–Do (TSD) approach alone. Group II: anesthesia procedures were combined with TSD and visual distraction using magic tricks (thumb sleeve). Group III: anesthesia procedures were combined with TSD and sensory distraction using a Pop‐It toy. Group IV: anesthesia procedures were combined with TSD and both visual (thumb sleeve) and sensory (Pop‐It toy) distractions.

Regarding physiological responses, pulse rate was assessed at three time points. At T0 (before treatment), median pulse rates ranged from 97 to 106 beats per minute, with mean values of 105.16 ± 13.88, 108.96 ± 15.80, 97.60 ± 13.99, and 99.44 ± 17.01 for Groups I, II, III, and IV, respectively. At T1 (after application of the behavior management technique), median pulse rates ranged from 98 to 108 beats per minute, with corresponding means of 105.40 ± 14.36, 104.12 ± 16.66, 97.32 ± 15.85, and 99.48 ± 17.13. At T3 (2 min after administration of local anesthesia), mean pulse rates were 110.76 ± 15.57, 111.44 ± 15.38, 109.28 ± 14.99, and 107.48 ± 16.45 for Groups I, II, III, and IV, respectively, with median values ranging from 107 to 110 beats per minute. Kruskal–Wallis testing showed no statistically significant differences in pulse rate among the study groups at any of the three time intervals (T0: *p*‐value = 0.074; T1: *p*‐value = 0.179; and T3: *p*‐value = 0.916) (Table 3).

**Table 3:** Descriptive statistics of children’s pulse rate across study groups at different time intervals and Kruskal–Wallis test results.

Studied groups	Median	Mean	Std. deviation	*p*‐Value
**T0 (before treatment)**				

Group I	106.00	105.16	13.883	0.074
Group II	106.00	108.96	15.802	
Group III	101.00	97.60	13.994	
Group IV	97.00	99.44	17.012	

**T1 (after application of the behavior management technique)**				

Group I	108.00	105.40	14.359	0.179
Group II	101.00	104.12	16.659	
Group III	100.00	97.32	15.853	
Group IV	98.00	99.48	17.130	

**T3 (after 2 min of administration of anesthesia)**				

Group I	108.00	110.76	15.573	0.916
Group II	110.00	111.44	15.379	
Group III	107.00	109.28	14.988	
Group IV	107.00	107.48	16.452	

*Note:* Group I (Control): anesthesia procedures were performed using the Tell–Show–Do (TSD) approach alone. Group II: anesthesia procedures were combined with TSD and visual distraction using magic tricks (thumb sleeve). Group III: anesthesia procedures were combined with TSD and sensory distraction using a Pop‐It toy. Group IV: anesthesia procedures were combined with TSD and both visual (thumb sleeve) and sensory (Pop‐It toy) distractions.

With respect to the Facial Image Scale [26], at T0, the mean values were 1.44 ± 0.65, 1.72 ± 1.31, 1.40 ± 0.71, and 1.24 ± 0.44 for Groups I, II, III, and IV, respectively. At T3, median scores remained 1.00 in all groups, with mean values of 1.56 ± 0.65, 1.48 ± 0.51, 1.72 ± 1.10, and 1.52 ± 0.77. Kruskal–Wallis analysis indicated no statistically significant differences in FIS scores among the groups either before treatment (*p*‐value = 0.663) or after anesthesia administration (*p*‐value = 0.968) (Table 4).

**Table 4:** Descriptive statistics of children’s Facial Image Scale scores across study groups and Kruskal–Wallis test results.

Studied groups	Median	Mean	Std. deviation	*p*‐Value
**T0 (before treatment)**				

Group I	1.00	1.44	0.651	0.663
Group II	1.00	1.72	1.308	
Group III	1.00	1.40	0.707	
Group IV	1.00	1.24	0.436	

**T3 (after 2 min of administration of anesthesia)**				

Group I	1.00	1.56	0.651	0.968
Group II	1.00	1.48	0.510	
Group III	1.00	1.72	1.100	
Group IV	1.00	1.52	0.770	

*Note:* Group I (Control): anesthesia procedures were performed using the Tell–Show–Do (TSD) approach alone. Group II: anesthesia procedures were combined with TSD and visual distraction using magic tricks (thumb sleeve). Group III: anesthesia procedures were combined with TSD and sensory distraction using a Pop‐It toy. Group IV: anesthesia procedures were combined with TSD and both visual (thumb sleeve) and sensory (Pop‐It toy) distractions.

Finally, the FLACC scale was used to assess behavioral responses during anesthesia administration (T2). The median scores were highest in Group I (3.00) and lowest in Group IV (0.00), while mean values were 3.16 ± 3.24, 1.84 ± 1.89, 2.36 ± 2.83, and 1.04 ± 1.40 for Groups I, II, III, and IV, respectively. Although lower scores were observed in the intervention groups compared with the control, the Kruskal–Wallis test showed no statistically significant differences among the groups (*p*‐value = 0.086) (Table 5).

**Table 5:** Descriptive statistics of children’s FLACC scale scores across study groups during anesthesia administration and Kruskal–Wallis test results.

Studied groups	Median	Mean	Std. deviation	*p*‐Value
**T2 (during administration of anesthesia)**				

Group I	3.00	3.16	3.236	0.086
Group II	1.00	1.84	1.886	
Group III	2.00	2.36	2.827	
Group IV	0.00	1.04	1.399	

*Note:* Group I (Control): anesthesia procedures were performed using the Tell–Show–Do (TSD) approach alone. Group II: anesthesia procedures were combined with TSD and visual distraction using magic tricks (thumb sleeve). Group III: anesthesia procedures were combined with TSD and sensory distraction using a Pop‐It toy. Group IV: anesthesia procedures were combined with TSD and both visual (thumb sleeve) and sensory (Pop‐It toy) distractions.

## 4. Discussion

Dental anxiety affects a significant proportion of pediatric patients, with prevalence estimates ranging from 6% to 20%, and higher rates are commonly reported among younger children [27]. As anxiety accounts for ~75% of routine dental care failures, its management is regarded as a critical component of pediatric dentistry, given that anxious children are often more difficult to treat and control [28].

Moreover, dental anxiety has been linked to reduced parental satisfaction with both planned and delivered treatments, which may, in turn, diminish perceptions of the dentist’s professional competence [29].

In addition, uncooperative behavior associated with anxiety can significantly compromise the delivery of high‐quality care by prolonging treatment duration, increasing the risk of injury to the child, and ultimately leading to heightened parental dissatisfaction [30].

Achmad’s 2021 systematic review identified three primary strategies for managing dental anxiety: nonpharmacological, pharmacological, and a combination of the two. This framework provides structured treatment options to support children experiencing anxiety during dental care, thereby serving as a potentially valuable guide in pediatric dentistry [31].

When a child’s anxiety is alleviated without the use of medication, the risks to their overall health are minimized, and access to urgent dental care is less likely to be delayed. Furthermore, this approach may enhance adherence to professional recommendations and preventive care in the future [32].

One effective method for reducing both dental pain and anxiety is the use of distraction techniques, which redirect the patient’s attention away from unpleasant procedures (noxious stimuli) by engaging their limited attention span [33].

These techniques are generally classified as either active or passive. Active strategies involve the child’s direct participation, such as playing games or using toys, whereas passive strategies rely on external stimuli, such as music, videos, or other media, and do not require active engagement from the child [34].

However, a review by Flores et al. [10] reported limited evidence supporting the effectiveness of distraction techniques. Further research comparing different distraction methods across age groups is therefore recommended to develop a clearer understanding of their impact and to inform their appropriate application in the dental setting.

Therefore, the current study aimed to evaluate the effectiveness of various distraction techniques in reducing dental fear, pain, and anxiety in children aged 4–6 years with no prior dental experience undergoing an IANB.

The 4–6‐year age group was selected due to the high prevalence of dental anxiety and pain observed in this population [35]. Furthermore, only children with no prior dental experience were included as previous dental encounters have been shown to influence the development and intensity of fear [36].

The IANB is the most commonly used and effective anesthetic technique for procedures involving primary dentition; however, it is also associated with the highest levels of discomfort and pain [37].

Pulse rate was measured as a dental anxiety index because stress, fear, anxiety, and pain can all induce an increase in heart rate, a response mediated by the sympathetic nervous system [38]. In addition, the Facial Image Scale was used because it is considered a validated self‐report scale that consists of five facial expressions, ranging from a very happy to a very sad face, designed to assess children’s levels of fear and anxiety. Scores range from 1 to 5, with 1 representing no anxiety (very happy face) and 5 indicating extreme anxiety (very sad face) [39, 40].

Finally, the FLACC scale is a reliable tool for use in pediatric clinical settings, demonstrating good validity and sensitivity in identifying pain in young children [41].

Regarding dental anxiety, no significant differences were observed between the groups in either pulse rate or Facial Image Scale scores. Nevertheless, Group I (TSD) consistently exhibited the highest scores across all measurement points, suggesting that while the differences were not statistically significant, this approach may elicit slightly higher observable anxiety responses in young children.

Moreover, the Kruskal–Wallis test revealed no significant differences between groups according to the FLACC scale following the administration of local anesthesia. However, lower scores were observed in the experimental groups compared with the control group, suggesting that combining the TSD technique with tools such as the thumb sleeve and Pop‐It may enhance the reduction of pain and distress in children.

Although the TSD technique and similar approaches are widely recognized as effective, they may not be suitable or acceptable for all children. Pharmacological interventions and physical restraints carry potential risks and may pose a threat to the child’s well‐being. For private practitioners, modeling and reinforcement strategies can be time‐consuming and, in some cases, ineffective. While aversive techniques may yield rapid results, changing attitudes among parents and dental professionals have led to a growing preference for nonaversive alternatives, such as distraction, which are increasingly valued for their safety and child‐centered approach [42].

Magic should be understood as a discipline that extends beyond tricks or illusions. Its effective performance requires preparation across three domains. Perception engages the senses and cognitive processes, drawing on attention, memory, and perceptual psychology. The dramatic dimension structures the performance through narrative elements, exposition, conflict, climax, and resolution, culminating in a compelling effect. Cognitive disruption, achieved through misdirection, manipulates the spectator’s logical and attentional processes while fostering a magical atmosphere that elevates the performance to an art form. Finally, the artistic dimension employs bodily expression, gaze, speech, and other expressive modalities to enhance both impact and aesthetic value [11].

A systematic review by Lekhwani et al. reported that the use of magic can serve as an effective tool for behavior management in anxious children undergoing dental treatment. This approach leverages engagement, distraction, and positive reinforcement to reduce fear and improve cooperation [15]. Furthermore, these findings are supported by multiple studies, which have consistently demonstrated that incorporating magic into pediatric dental practice can enhance the overall patient experience, reduce procedural anxiety, and facilitate treatment compliance [12–14].

Thumb sleeves incorporating a lighting device can be used as a thaumaturgic tool in pediatric dental practice. The device is worn on the operator’s thumb and can be activated or deactivated at will, allowing for a range of creative hand movements that capture and sustain a child’s attention. This approach is particularly effective in children aged 2–7 years as the intermittent appearance and disappearance of light is perceived as a magical phenomenon, enhancing engagement and reducing anxiety during dental procedures [30].

Although this study did not reveal significant differences compared with the TSD technique alone, children exposed to magic tricks recorded lower dental anxiety and pain scores. These findings align with Kothari et al. [12], who reported similar outcomes among children aged 4–6 years requiring IANB, although their study identified significant differences between the thaumaturgic aid and euphemistic communication, likely due to variations in assessment tools.

Thosar et al. [43] also demonstrated comparable effects of magic tricks and audio–visual distraction in 30 children aged 4–11 years undergoing restorative treatment, with discrepancies from our results potentially attributable to differences in comparison groups, age ranges, outcome measures, and procedures. Likewise, Shree et al. [13] reported that magic tricks produced the significantly lowest anxiety scores among 60 children aged 4–13 years receiving orthodontic or surgical treatment under local anesthesia, highlighting the influence of procedural and methodological differences.

Konde et al. [14] further confirmed the value of thaumaturgy in behavior management among 240 children aged 2–13 years, whereas Asokan et al. [44] found that TSD, mobile dental games, and magic tricks were equally effective in reducing pain and anxiety in 60 children aged 4–5 years undergoing scaling. Variations in study design, outcome measures, and treatment procedures may account for these differences.

The calming effect of magic may stem from activation of the right cerebral hemisphere, which is central to perceiving magical phenomena and is more developed in children aged 3–6 years, making them especially receptive to imaginative engagement. In some cases, magic may also stimulate curiosity and cognitive processing, thereby involving the left hemisphere [45].

Therapeutic play is considered an effective method for reducing anxiety in children during medical and dental procedures. It can help alleviate negative emotions such as anger, hostility, worry, and fear, providing a safe outlet for expression. By engaging in play, children may develop coping strategies that enable them to manage anxiety more effectively during treatment [46].

Pop‐It therapeutic play uses silicone Pop‐It toys—pressable bubble‐like structures available in various shapes, colors, and sizes—to promote relaxation and support children’s motor, sensory, and cognitive development. Beyond their recreational appeal, these toys enhance fine motor coordination and sensory processing. Their widespread popularity, amplified by social media, makes them highly accessible, and their engaging, calming nature suggests potential as a nonpharmacological tool to reduce anxiety and improve cooperation in pediatric dental procedures [16].

Evidence from medical settings suggests that Pop‐It‐based therapeutic play interventions may have beneficial effects in reducing anxiety; however, their application in pediatric dental settings has been minimally investigated [16].

Various types of games, including mobile applications [47], dental instrument toys, and cartoon characters [48], have been used to reduce dental pain and anxiety in children. A systematic review by Martos et al. [49] concluded that game‐based interventions effectively alleviate preoperative anxiety during anesthesia induction in pediatric patients. This innovative and engaging approach may hold promise for improving the management of anxiety in pediatric dental care.

Play therapy is effective through six key components: the therapeutic relationship, diagnostic opportunities, breaking down defense mechanisms, facilitating expression, therapeutic release, and anticipatory preparation. It addresses cognitive, affective, and interpersonal domains, enabling practitioners to modify beliefs, regulate emotions, and strengthen social support through structured play. Sessions should be tailored flexibly to meet specific therapeutic goals [50].

This study has several limitations. Participants had no prior dental experience, restricting assessment of behavior influenced by previous exposure, and only children demonstrating positive behavior, as measured by the Frankl behavior rating scale, were included, potentially affecting the observed outcomes. Additional limitations include the short‐term follow‐up, which precludes the evaluation of long‐term behavioral changes. Furthermore, behavioral assessments relied primarily on subjective measures and incorporated only one physiological indicator of anxiety; the inclusion of additional objective measures, such as salivary cortisol or electrodermal activity, could have provided a more comprehensive evaluation. Finally, no subjective pain assessment was used, which limits the understanding of children’s perceptions of the procedures.

## 5. Conclusion

Considering the limitations of this study, although no significant differences were observed between visual distraction, sensory distraction, and the TSD technique alone, the use of distraction appeared to enhance the effectiveness of TSD in reducing dental pain and anxiety. Therefore, integrating these inexpensive, readily available, and easy‐to‐use tools into routine dental procedures may improve clinical efficiency and performance while fostering a positive attitude in children during dental visits, especially those aged 4–6 years, during an IANB procedure.

**Nomenclature**
TSD: Tell–Show–Do
FI: facial image
FLACC: face, legs, activity, cry, consolability
IANB: inferior alveolar nerve block.

## Author Contributions

**Alaa Mohammed Snobar:** research concept and design, collected data, extracted the data, contributed to writing. **Chaza Nader Kouchaji**: research concept and design, supervised the project, performed critical revision of the manuscript. **Mohammed N. Al-Shiekh**: performed the statistical analysis, contributed to writing.

## Funding

The authors have nothing to report.

## Disclosure

All authors have read and approved the manuscript.

## Ethics Statement

This study was conducted in accordance with the ethical principles outlined in the Declaration of Helsinki and adhered to the CONSORT guidelines [18, 19]. The Ethics Committee of Damascus University approved this study (Approval Number 3176/22042024).

## Conflicts of Interest

The authors declare no conflicts of interest.

## Data Availability

The datasets generated during and/or analyzed during the current study are available from the corresponding author upon reasonable request.

## References

[1] Menni A. C. , Radhakrishna A. N. , and Prasad M. G. , DentalVibe Versus Lignocaine Hydrochloride 2% Gel in Pain Reduction During Inferior Alveolar Nerve Block in Children, Journal of Dental Anesthesia and Pain Medicine. (2020) 20, no. 6, 10.17245/jdapm.2020.20.6.397, 397. 33409368 PMC7783374

[2] Tarrosh M. Y. , Alhazmi Y. A. , and Aljabri M. Y. , et al.A Systematic Review of Cross-Sectional Studies Conducted in the Kingdom of Saudi Arabia on Levels of Dental Anxiety Between Genders and Demographic Groups, Medical Science Monitor: International Medical Journal of Experimental and Clinical Research. (2022) 28, e937470–e937471, 10.12659/MSM.937470. 35908171 PMC9351396

[3] Grisolia B. M. , dos Santos A. P. P. , Dhyppolito I. M. , Buchanan H. , Hill K. , and Oliveira B. H. , Prevalence of Dental Anxiety in Children and Adolescents Globally: A Systematic Review With Meta-Analyses, International Journal of Paediatric Dentistry. (2021) 31, no. 2, 168–183, 10.1111/ipd.12712. 33245591

[4] Almarzouq S. S. , Chua H. , Yiu C. K. , and Lam P. P. , Effectiveness of Nonpharmacological Behavioural Interventions in Managing Dental Fear and Anxiety Among Children: A Systematic Review and Meta-Analysis, Healthcare. (2024) 12, no. 5, 537. 38470648 10.3390/healthcare12050537 PMC10931341

[5] Del Carmen M. D. C. , Cagigas-Muñiz D. , García-Robles R. , and Oprescu A. M. , Reducing Dental Anxiety in Children Using a Mobile Health App: Usability and User Experience Study, JMIR Formative Research. (2023) 7, 10.2196/30443, e30443. 37889521 PMC10638634

[6] Raja S. N. , Carr D. B. , and Cohen M. , et al.The Revised International Association for the Study of Pain Definition of Pain: Concepts, Challenges, and Compromises, Pain. (2020) 161, no. 9, 1976–1982, 10.1097/j.pain.0000000000001939. 32694387 PMC7680716

[7] American Academy of Pediatric Dentistry (AAPD), Council on Clinical Affairs , Behavior Guidance for the Pediatric Dental Patient, Pediatric Dentistry. (2018) 40, no. 6, 254–267. 32074897

[8] Qureshi R. , Iqbal A. , and Khan M. , et al.Assessment of Parental Acceptance Towards Different Non‑Pharmacological Behaviour Management Techniques in Pediatric Dental Care — A Cross‑Sectional Study, Journal of Clinical Pediatric Dentistry. (2023) 47, no. 4, 35–39, 10.22514/jocpd.2023.033. 37408344

[9] Alsibai E. , Bshara N. , Alzoubi H. , and Alsabek L. , Assessing an Active Distracting Technique During Primary Mandibular Molar Pulpotomy (Randomized Controlled Trial), Clinical and Experimental Dental Research. (2023) 9, no. 2, 283–289, 10.1002/cre2.702. 36478192 PMC10098273

[10] Flores A. M. A. , Gómez M. R. , and González G. , et al.Distraction Techniques in Children With Dental Fear and Anxiety, International Journal of Applied Dental Sciences. (2022) 8, no. 1, 513–516, 10.22271/oral.2022.v8.i1h.1469.

[11] Villamizar J. , Use of Magic as an Alternative in Behavioral Management in Pediatric Dentistry, Advances in Dentistry and Oral Health. (2023) 16, no. 4, 555942.

[12] Kothari P. , Mathur A. , Chauhan R. S. , Nankar M. , Tirupathi S. , and Suvarna A. , Effectiveness of Thaumaturgic Distraction in Alleviation of Anxiety in 4-6-Year-Old Children During Inferior Alveolar Nerve Block Administration: A Randomized Controlled Trial, Journal of Dental Anesthesia and Pain Medicine. (2023) 23, no. 3, 10.17245/jdapm.2023.23.3.143, 143. 37313267 PMC10260356

[13] Nagar P. , Mascarenhas A. N. , Pooja H. R. , and Shree C. H. C. K. , Magic: A Modern Alleviating Constituent of Anxiety Levels in Children, Journal of South Asian Association of Pediatric Dentistry. (2022) 5, no. 3, 121–126, 10.5005/jp-journals-10077-3241.

[14] Agarwal M. , Konde S. , Sumaiyya S. , and Peethambar P. , “Thaumaturgy”—A Novel Behavior‑Shaping Technique, International Journal of Clinical Pediatric Dentistry. (2020) 13, no. 4, 318–321, 10.5005/jp-journals-10005-1781. 33149401 PMC7586480

[15] Tirupathi S. and Afnan L. , Thaumaturgical Distraction as a Modality for Reducing Dental Anxiety in Children: A Systematic Review, International Journal of Clinical Pediatric Dentistry. (2024) 17, no. 11, 1296–1301, 10.5005/jp-journals-10005-2998. 39781396 PMC11703761

[16] Bawaeda O. , Wanda D. , and Aprillia Z. , Effectiveness of Pop‑It Therapeutic Play on Children’s Anxiety During Inhalation Therapy in Children’s Wards, La Pediatria Medica e Chirurgica. (2023) 45, no. s1, 10.4081/pmc.2023.315, 315. 36974915

[17] Mendri N. K. , Badi A. , and Najib M. , Pop Up Toys as Story Play Therapy on the Level of Anxiety on General Anesthesia Surgery Among Children Around 6–12 Years Old, *7th International Conference on Public Health (ICPH)*, 2020, Surakarta, Indonesia, Sebelas Maret University.

[18] Cuschieri S. , The CONSORT Statement, Saudi Journal of Anaesthesia. (2019) 13, no. 5, S27–S30, 10.4103/sja.SJA_559_18. 30930716 PMC6398298

[19] Shrestha B. and Dunn L. , The Declaration of Helsinki on Medical Research involving Human Subjects: A Review of Seventh Revision, Journal of Nepal Health Research Council. (2020) 17, no. 4, 548–552, 10.33314/jnhrc.v17i4.1042. 32001865

[20] Gupta K. K. , Attri J. P. , Singh A. , Kaur H. , and Kaur G. , Basic Concepts for Sample Size Calculation: Critical Step for Any Clinical Trials!, Saudi Journal of Anaesthesia. (2016) 10, no. 3, 328–331, 10.4103/1658-354X.174918. 27375390 PMC4916819

[21] Miller J. , Ulrich R. , and Li Y. , The Quest for an Optimal Alpha, PLoS ONE. (2019) 14, no. 1, 10.1371/journal.pone.0208631, e0208631. 30601826 PMC6314595

[22] Fritz C. O. , Morris P. E. , and Richler J. J. , Effect Size Estimates: Current use, Calculations, and Interpretation, Journal of Experimental Psychology: General. (2012) 141, no. 1, 2–18, 10.1037/a0024338. 21823805

[23] Crellin D. J. , Harrison D. , Santamaria N. , and Babl F. E. , Systematic Review of the Face, Legs, Activity, Cry and Consolability Scale for Assessing Pain in Infants and Children: Is It Reliable, Valid, and Feasible for Use?, Pain. (2015) 156, no. 11, 2132–2151, 10.1097/j.pain.0000000000000305. 26207651

[24] Peng T. , Qu S. , Du Z. , Chen Z. , Xiao T. , and Chen R. , A Systematic Review of the Measurement Properties of Face, Legs, Activity, Cry and Consolability Scale for Pediatric Pain Assessment, Journal of Pain Research. (2023) 16, 1185–1196, 10.2147/JPR.S397064. 37064956 PMC10094406

[25] Alkhouli M. , Al-Nerabieah Z. , and Dashash M. , Can Computerized Intraosseous Anaesthesia Replaces the Inferior Alveolar Nerve Block in Children? A Randomized Controlled Clinical Trial, Oral Surgery. (2025) 18, no. 2, 145–151, 10.1111/ors.12930.

[26] Stawarczyk B. , Ender A. , Trottmann A. , Özcan M. , Fischer J. , and Hämmerle C. H. F. , Load-Bearing Capacity of CAD/CAM Milled Polymeric Three-Unit Fixed Dental Prostheses: Effect of Aging Regimens, Clinical Oral Investigations. (2012) 16, no. 6, 1669–1677, 10.1007/s00784-011-0670-4. 22209963

[27] Appukuttan D. , Strategies to Manage Patients With Dental Anxiety and Dental Phobia: Literature Review, Clinical, Cosmetic and Investigational Dentistry. (2016) 8, 35–50, 10.2147/CCIDE.S63626. 27022303 PMC4790493

[28] Cajares C. M. , Rutledge C. M. , and Haney T. S. , Animal Assisted Therapy in a Special Needs Dental Practice: An Interprofessional Model for Anxiety Reduction, Journal of Intellectual Disability - Diagnosis and Treatment. (2016) 4, no. 1, 25–28, 10.6000/2292-2598.2016.04.01.3.

[29] Viswanath D. , Kumar M. , and Prabhuji M. , Dental Anxiety, Fear and Phobia in Children, International Journal of Dental Research and Development. (2014) 4, no. 1, 1–14.

[30] Fatma N. , Contemporary Distraction Tools Used in Pediatric Dentistry: An Overview, University Journal of Dental Sciences. (2021) 7, no. 3, 28.

[31] Achmad H. , Management of Pediatric Patients with Anxiety on Dental Care: A Systematic Review, Annals of the Romanian Society for Cell Biology. (2021) 25, no. 2, 1132.

[32] Roberts J. F. , Curzon M. E. J. , Koch G. , and Martens L. C. , Behaviour Management Techniques in Paediatric Dentistry, European Archives of Paediatric Dentistry. (2010) 11, no. 4, 166–174, 10.1007/BF03262738. 20840826

[33] Al-Jaloud M. M. , Al-Osaidi K. S. , and Al-Anzi S. S. , et al.Effect of Various Distraction Techniques On Pain and Anxiety of Pediatric Dental Patients: A Systematic Review, Pharmacophore. (2022) 13, no. 5, 105–111, 10.51847/vTfTdjBaws.

[34] Nuvvula S. , Alahari S. , Kamatham R. , and Challa R. R. , Effect of Audiovisual Distraction With 3D Video Glasses On Dental Anxiety of Children Experiencing Administration of Local Analgesia: A Randomised Clinical Trial, European Archives of Paediatric Dentistry. (2015) 16, no. 1, 43–50, 10.1007/s40368-014-0145-9. 25256207

[35] Majstorovic M. and Veerkamp J. S. , Developmental Changes in Dental Anxiety in a Normative Population of Dutch Children, European Journal of Paediatric Dentistry. (2005) 6, no. 1, 30–34. 15839831

[36] Suprabha B. S. , Rao A. , Choudhary S. , and Shenoy R. , Child Dental Fear and Behavior: The Role of Environmental Factors in a Hospital Cohort, Journal of Indian Society of Pedodontics and Preventive Dentistry. (2011) 29, no. 2, 95–101, 10.4103/0970-4388.84679. 21911945

[37] Gajendragadkar K. , Bhate K. , Jagtap B. , S.N S. , Kshirsagar K. , and Magoo S. , Making Inferior Alveolar Nerve Block More Comfortable via Computer-Controlled Local Anesthetic Delivery: A Prospective Clinical Study, Journal of Dental Anesthesia and Pain Medicine. (2019) 19, no. 3, 135–141, 10.17245/jdapm.2019.19.3.135. 31338419 PMC6620535

[38] Alkanan S. A. M. , Alhaweri H. S. , Khalifa G. A. , and Ata S. M. S. , Dental Pain Perception and Emotional Changes: On the Relationship Between Dental Anxiety and Olfaction, BMC Oral Health. (2023) 23, no. 1, 10.1186/s12903-023-02864-9, 175. 36966288 PMC10040111

[39] Yon M. J. Y. , Chen K. J. , Gao S. S. , Duangthip D. , Lo E. C. M. , and Chu C. H. , An Introduction to Assessing Dental Fear and Anxiety in Children, Healthcare. (2020) 8, no. 3, 263. 32260395 10.3390/healthcare8020086 PMC7348974

[40] Fathima F. and Jeevanandan G. , Validation of a Facial Image Scale to Assess Child Dental Anxiety, Drug Invention Today. (2018) 10, no. 1, 2825–2829.

[41] Felemban O. M. , Alshamrani R. M. , Aljeddawi D. H. , and Bagher S. M. , Effect of Virtual Reality Distraction On Pain and Anxiety During Infiltration Anesthesia in Pediatric Patients: A Randomized Clinical Trial, BMC Oral Health. (2021) 21, no. 1, 10.1186/s12903-021-01678-x, 321. 34172032 PMC8234622

[42] Navit S. , Effectiveness and Comparison of Various Audio Distraction Aids in Management of Anxious Dental Paediatric Patients, Journal of Clinical and Diagnostic Research. (2015) 9, no. 12, 10.7860/JCDR/2015/15564.6910, ZC05. PMC4717707 26816984

[43] Thosar N. R. , Bane S. P. , Deulkar P. V. , Deshpande M. A. , and Gupta S. , Effectiveness of Two Different Behavior Modification Techniques for Anxiety Reduction in Children, Cureus. (2022) 14, no. 8, 10.7759/cureus.28141, e27856. 36134077 PMC9482440

[44] Asokan S. , Geetha Priya P. R. , Natchiyar S. N.ambi , and Elamathe M. , Effectiveness of Distraction Techniques in the Management of Anxious Children–A Randomized Controlled Pilot Trial, Journal of Indian Society of Pedodontics and Preventive Dentistry. (2020) 38, no. 4, 407–412, 10.4103/JISPPD.JISPPD_435_20. 33402625

[45] Peretz B. and Gluck G. , Magic Trick: A Behavioural Strategy for the Management of Strong-Willed Children, International Journal of Paediatric Dentistry. (2005) 15, no. 6, 429–436, 10.1111/j.1365-263X.2005.00668.x. 16238653

[46] Sezici E. , Ocakci A. F. , and Kadioglu H. , Use of Play Therapy in Nursing Process: A Prospective Randomized Controlled Study, Journal of Nursing Scholarship. (2017) 49, no. 2, 162–169, 10.1111/jnu.12277. 28098954

[47] Karkoutly M. , Al-Halabi M. N. , Laflouf M. , and Bshara N. , Effectiveness of a Dental Simulation Game On Reducing Pain and Anxiety during Primary Molars Pulpotomy Compared With Tell-Show-Do Technique in Pediatric Patients: A Randomized Clinical Trial, BMC Oral Health. (2024) 24, no. 1, 10.1186/s12903-024-04732-6, 976. 39174937 PMC11342516

[48] Naik V. , Nayak P. P. , and Bhatia K. , et al.Feasibility of Tell-Play-Do Intervention in Outreach Programs: A Group-Based Behavior Management Approach for Anxiety Reduction, International Journal of Clinical Pediatric Dentistry. (2025) 18, no. 6, 688–694, 10.5005/jp-journals-10005-3152. 41041003 PMC12486470

[49] Suleiman-Martos N. , García-Lara R. A. , and Membrive-Jiménez M. J. , et al.Effect of a Game-Based Intervention On Preoperative Pain and Anxiety in Children: A Systematic Review and Meta-Analysis, Journal of Clinical Nursing. (2022) 31, no. 23-24, 3350–3367, 10.1111/jocn.16227. 35075716 PMC9787560

[50] Godino-Iáñez M. J. , Martos-Cabrera M. B. , and Suleiman-Martos N. , et al.Play Therapy as an Intervention in Hospitalized Children: A Systematic Review, Healthcare. (2020) 8, no. 4, 353. 32751225 10.3390/healthcare8030239 PMC7551498

